# Sustaining temporal attention prevents habit expression during operant learning in rats

**DOI:** 10.1038/s41598-020-67304-y

**Published:** 2020-06-25

**Authors:** Ziqiao Lin, Hiromi Nishikawa, Yoshio Iguchi, Akira Iwanami, Mitsuru Kikuchi, Shigenobu Toda

**Affiliations:** 1 Department of Psychiatry and Behavioral Science, Kanazawa University School of Medicine, Kanazawa, Japan; 2 Research Center for Child Mental Development, Kanazawa University, Kanazawa, Japan; 3 Department of Molecular Genetics, Institute of Biomedical Sciences, Fukushima Medical University, Fukushima, Japan; 4 Department of Psychiatry, Showa University School of Medicine, Shinagawa, Japan

**Keywords:** Attention, Decision, Basal ganglia

## Abstract

As repeated operant performance promotes the transition from goal-directed action to habitual response, it appears that action-outcome contingency learning precedes and is necessary for the transition. Meanwhile, it is known that operant performance under a fixed interval (FI) schedule, in which the timing of reinforcement is predictable, is resistant to habit. However, the reason why the FI schedule prevents habit expression remains unclear. We reasoned that sustained attention for monitoring a certain interval might require a goal-directed process and prevent the transition. To verify this hypothesis, rats underwent FI schedule operant training while auditory cues were provided in a manner either contingent or non-contingent with the timing of lever pressing to obtain a reward. The subjects developed a habit with contingent cues, but not with either non-contingent cues or no cues. Overall, we conclude that the release from sustained attentional burden allows the expression of habit. (147 words)

## Introduction

When an action is performed repeatedly, it will eventually become habitual. Forming habits is essential in life, enabling almost automatic and prompt output and the ability to multitask with minimal use of attention and motivation^1^. In general, the instrumental performance is goal-directed at a pre-habitual stage. A goal-directed action is inferred as sensitivity to either outcome devaluation or contingency degradation, whereas, a habitual response is regarded to be insensitive to both^2^. At the goal-directed stage, a subject attempts to learn the association (contingency) of action (A) with its outcome (O), known as A–O contingency. In both rodents and humans, this associative learning is acquired through the dorsomedial striatum and cortical areas that project into the striatal subregion^3,4^. Lesioning these brain regions not only impairs A–O contingency learning but also promotes habitual action, even with fewer training sessions^3^. Thus, the A–O contingency learning seems to be a rate-limiting factor for putting off the transition from goal-directed action to the habitual response.

Meanwhile, it is also known that some conditions regarding operant performance are habit-prone, whereas others are not. For example, under a random interval (RI) schedule (e.g., RI60-s; a rewarding outcome is given randomly in each period of 60 s), it is difficult to predict the timing of reinforcement. However, action can be habitual under this schedule. Conversely, under a fixed interval (FI) schedule (e.g., FI60-s; a reward is given every 60 s by lever pressing), animals can predict the timing of rewards. Nevertheless, in the FI schedule, the transition from goal-directed to habitual action is more difficult than it is in the RI schedule^5^. DeRusso and colleagues have argued that higher uncertainty induced by the lower A-O contiguity in the RI schedule compared with that in the FI schedule may be responsible for being habit-prone^5^. Meanwhile, a recent paper by Thrailkill and colleagues has suggested that in the RI schedule subjects stop attending to their actions when it can to predict outcome availability by presenting a discriminative stimulus, which may promote habits even in the RI schedule^6^. Then, what would happen if the necessity to attend their actions or anything else is manipulatively attenuated in the FI schedule? Does it affect the goal-directed nature of the FI schedule?

To address these issues, we focused on sustained attention for estimating a designated elapsed interval under the FI schedule, and we hypothesized that it may prevent action from becoming habitual. We then investigated what might happen to the transition if this attentional burden is removed from the FI schedule by adding an auditory cue signaling the end of the required delay of reinforcement. We anticipated that, once a subject is released from the attentional burden, their action would become habitual, even under the FI schedule, and that the presence of attentional (cognitive) burden is a rate-limiting factor for the transition from goal-directed action to the habitual response.

## Materials and Methods

### Animals and apparatus

Sprague–Dawley male rats weighing 275–300 g (Japan SLC, Hamamatsu, Japan) were housed individually and maintained under a 12-h light-dark cycle (lights on at 08:45 h). All procedures were performed following the Guidelines for Proper Conduct of Animal Experiments (Science Council of Japan, June 2006) and were approved by the Institutional Animal Care and Use Committee of Kanazawa University. Behavioral training and testing were performed in standard operant chambers (Med Associates, St. Albans, VT, USA) equipped with a sonalert (ENV-233AM; Med Associates) that provided a 2,900 Hz, 65 dB of tone. Each chamber contained a recessed food magazine from which 45-mg food pellets (F0021; Bioserv, Flemington, NJ, USA) were delivered as a reinforcing outcome, and each magazine had one retractable lever on the left-hand side.

### Interval schedules

During an FI schedule, the first lever press was rewarded only once a fixed amount of time had elapsed since the previous reinforcement. Also, the device initiated a time-out when the lever press input was not received within a specified period (10 s, 30 s, or 60 s after a fixed interval). (e.g., time-out = 10 s means that a food pellet will be dispensed immediately after a lever press only if the rats press the lever within the 10 s after the fixed interval).

### Lever-press training

Initial training consisted of five daily 30 min sessions: apparatus habituation, magazine training (during which outcome pellets were delivered at random in a 30 s schedule), manual shaping for a left lever with 60 outcomes, and continuous reinforcement training (CRF) (two sessions)^5^.

Following two CRF sessions, the rats were trained for three days on an FI 5-s schedule (a food pellet reward for the first lever press 5 s after the previous reinforcement; the time-out was 60 s), followed by three days on a FI 10-s schedule (time-out = 60 s), three days on an FI 20-s schedule (time-out = 60 s, 30 s, and 10 s, reduced), three days on an FI 30-s schedule (time-out: same as above), and, finally, three days on an FI 45-s schedule (time-out: same as above). Then, the rats were trained for two days on an FI 60-s schedule (time-out = 60 s and 30 s, reduced in a stepwise manner). Then, lever pressing was reinforced during an FI 60-s schedule (time-out = 10 s) lasting 14 days.

**Figure 1:**
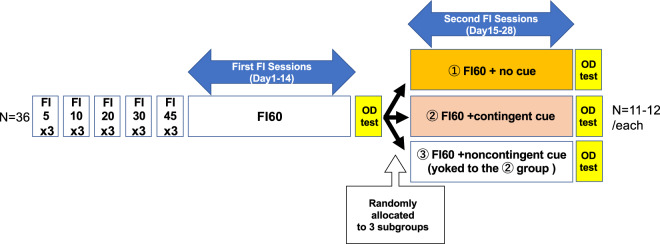
A scheme of experimental design for the present study.

The animals were then assigned randomly to three groups: FI + no cue, FI + contingent cue, and FI + non-contingent cue (N = 12/each; Fig. 1). Each group subsequently underwent 14 training sessions of reinforcement on a FI 60-s schedule (time-out = 10 s) under different conditions. A toner was presented for the FI + contingent cue group animals immediately after 60 s had elapsed since the previous reinforcement (= contingent cue). Each contingent cue persisted either until the animal responded or until after 10 s had elapsed; this was set as the time-out. The same tone was provided to each animal in the FI + non-contingent cue group, but in a yoked manner to the animals in the FI + contingent cue group, so that the auditory stimulus did not inform the reward timing in this group. Animals in the FI + no cue group were trained without any auditory cue. Each lever press training session lasted 30 min. During training and testing, each session began with a lever presentation after a waiting period of 300 s without either stimuli or lever presentations.

### Outcome devaluation test

The animals’ sensitivity to outcome devaluation was assessed in a two-day test under both devalued and non-devalued conditions (order counterbalanced), as conducted previously^7^. In brief, for the devalued condition, each rat was provided with 30 min *ad libitum* access to the training outcome in individual consumption cages. For the non-devalued condition, each animal was provided with 30 min *ad libitum* access to lab chow. Immediately after these satiety manipulations, the animals were placed in the operant chambers, where their responses to the training lever were tested for 5 min without food delivery (i.e., on extinction). The duration of *ad libitum* access to food and the time-bin were chosen according to previous studies^8,9^. The researcher who conducted the test confirmed that the rate of food consumption became almost null at the end of the devaluation test sessions (not objectively quantified). All three groups were tested simultaneously, by allocating each group into three subgroups. The first outcome devaluation test was conducted after 14 days of training on the FI60 schedule. The second outcome devaluation test was conducted in the same way following 14 further FI training sessions with three distinct conditions, as described above (Fig. 1).

### Statistical analyses

All data were statistically analyzed using one-way or two-way analysis of variance (ANOVA) conducted in Prism8, ANOVA4 (https://www.hju.ac.jp/kiriki/anova4/), and Microsoft Excel. We conducted two-way ANOVAs for each group to compare between devalued and non-devalued conditions [*Test* (2: before and after the second FI sessions) × *Devaluation* (2: devalued vs. non-devalued)]. For employing two-way ANOVAs for testing the effect of devaluation test on each group, the values were normalized using the response number on the previous day of the test session during the corresponding first 5 min period to obtain normally distributed values. Based on the assumption that the sensitivity to the second outcome devaluation test can be compromised in the FI + contingent group, we continued to conduct a simple main effect analysis. The effect size and its confidence interval were calculated using the Effect Size Calculators (see https://effect-size-calculator.herokuapp.com/#form4). The reliability of the result was assessed against a type I error (*α*) of 0.05. In the analyses of outcome devaluation tests, data exceeding the mean ± 3 SD in each group were excluded from the analyses as outliers.

## Results

**Figure 2:**
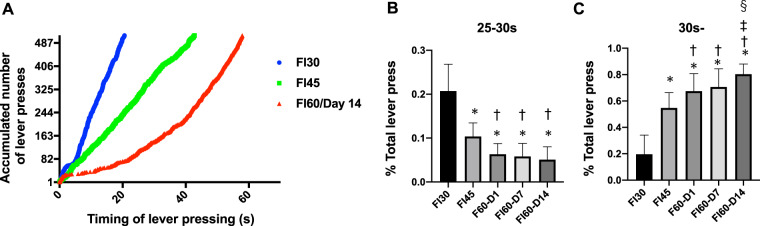
(**A**) A representative development of the lever press based on an operant learning from FI 30-s, FI 45-s to FI 60-s (Day 14) in an individual rat. X-axis depicts the timing of lever press, whereas Y-axis depicts the accumulated number of lever press (seconds). Dot lines present the designated timings at which subjects ought to press the levers under each FI schedule. (**B**,**C**). The percentage of the lever press during the designated period (B; 25–30 s, C; 30 s-) in the total number of lever press. Bar: mean ± S.D. In panel B, *indicates significant differences v.s. FI 30-s, ^†^indicates significant differences v.s. FI 45-s (*ps* < 0.01). In panel C, *indicates significant differences v.s. FI 30-s ^†^indicates significant differences v.s. FI 45-s, ^‡^indicates significant differences v.s. D1 of FI 60-s and ^§^indicates a significant difference v.s. D7 of FI 60-s (*ps* < 0.01).

Initially, we tried to train rats via operant learning under an FI schedule. Animals were trained under different FI schedules, from the easiest, i.e., FI 5-s, to FI 10, FI 15, FI 20, FI 30, FI 45, and, finally, FI 60-s, in order of interval length. Figure 2A shows the development of an individual animal’s lever pressing according to the progress of the FI schedule, from shorter intervals to the longest. The average number of lever press between 25–30 s gradually decreased as the FI sessions developed. This observation was supported by a one-way ANOVA for the average number of lever press by *session timepoint* (FI 30-s, FI 45-s, Day1, Day7, and Day14 of FI60-s). There was a significant main effect of *session timepoint* (*F*(4, 175) = 108.3, *p* < 0.01, partial **η**^2^ = 0.71, 95% confidence interval [CI] = [0.63, 0.75]), and *post hoc* tests indicated that the differences between FI 30-s or FI 45-s and other later dates were all significant (*ps* < 0.01; Fig. 2B). Conversely, the one after 30 s increased. A one-way ANOVA for the average number of lever press by *session timepoint* (FI 30-s, FI 45-s, Day1, Day7, and Day14 of FI 60-s) found a significant main effect of *session timepoint* (*F*(4, 175) = 131.1, *p* < 0.01, partial **η**^2^ = 0.75, 95% CI = [0.68, 0.78]) and *post hoc* Bonferroni tests indicate that the differences between FI 30-s or FI 45-s and other later dates as well as the ones between Day14 of FI 60-s and two previous dates were all significant (*ps* < 0.01; Fig. 2C). Also, the median of the timing of lever pressing increased gradually from FI 30-s to FI 45-s and FI 60-s (Supp Fig. 1A; *see* supplemental information) whereas the proportion of lever press at 25–30 s in total, that is the marker of the achievement of FI 30-s, was decreased at the same time (Supp Fig. 1B; supplemental information). Similarly, as the operant training under the FI 60-s schedule proceeded, the number of lever presses at a shorter interval (25–30 s) reduced, whereas the number of lever presses at longer intervals (40–45 s and 55–60 s) increased (Supp Fig. 2; supplemental information). Overall, we concluded that the animals increased the rate of their lever pressing toward the designated timing by adapting the shift from shorter FIs to the longest FI in a stepwise manner, as anticipated.

We then examined the nature of the acquired FI 60-s schedule. As shown in Supp Fig. 3, the number of lever presses, the number of pellets obtained, and the ratio of lever presses to pellets were left unchanged during the first 14 days of training, indicating that repeated training had no significant effect on the number of lever presses, the number of pellets obtained, and the efficiency of the rats’ actions (supplemental information).

Then, to investigate the effect of contingent or non-contingent auditory cues on habit expression under the FI schedule, we divided this cohort at random into three subgroups: FI + no cue, FI + non-contingent cue, and FI + contingent cue. During the following second 14-day period of each training schedule, there were again no significant differences between the three groups in terms of the number of lever presses, the number of pellets obtained, and the ratio of lever presses to pellets, except a few (Supp Fig. 4; supplemental information).

**Figure 3:**
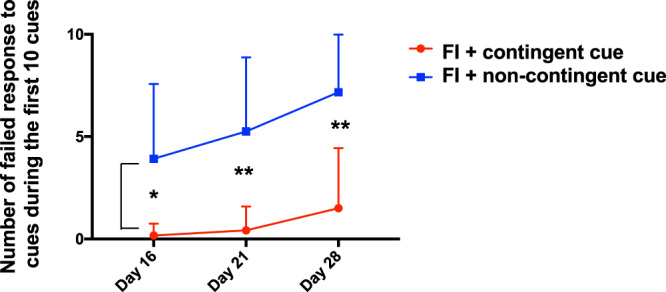
Comparison of the number of omitted responses to cues between FI + contingent cue group and FI + non-contingent cue group. N = 12/each. Bar: mean ± S.D. *p < 0.05, **p < 0.01.

The timings of the auditory cues presented to both contingent- and non-contingent groups should be the same owing to the nature of the yoked procedure. However, as the reaction to the cues is occasionally and/or accidentally skipped, the timing of reinforcement toward the cue presentation will become different little by little between the two groups. In the contingent group, all reinforcement should occur during the cue presentations. On the contrary, the contingency between the cues and the reward timing in the non-contingent group should be gradually deteriorated, which makes the animals learn that these cues are irrelevant. As a result, we speculate that this group will start to ignore the cues. As expected, compared to the FI + contingent cue group, the FI + non-contingent group displayed a robust increase in the number of omissions to cues (two-way ANOVA, *Group* (2: FI + contingent cue, FI + non-contingent cue) × *Days* (3: Day 16, Day 21, Day 28); *interaction*, *F*(2, 66) = 0.7454, *p* = 0.48; *Day*, *F*(2, 66) = 4.352, *p* = 0.02, partial **η**^2^ = 0.12, 95% CI = [0, 0.25]; *Group, F*(1, 66) = 54.39, *p* < 0.01, partial **η**^2^ = 0.62, 95% CI = [0.46, 0.71]; for subsequent tests, *see* supplemental information), indicating that this group developed indifference to irrelevant cues, and the yoked group worked as another negative control (Fig. 3).

**Figure 4:**
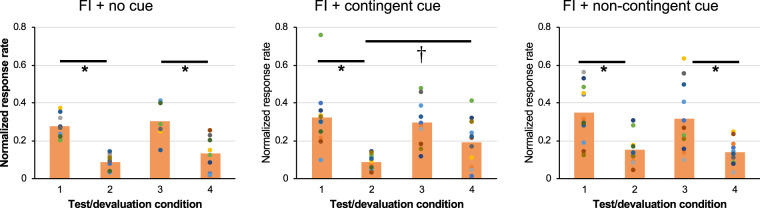
Comparisons of the sensitivity of outcome devaluation tests of each FI training between before and after the second FI sessions. In X-axis, bars 1–4 demonstrate 1^st^ devaluation test before second FI sessions/non-devalued condition (bar 1), 1^st^ devaluation test before second FI sessions/devalued condition (bar 2), 2^nd^ devaluation test after second FI sessions/non-devalued condition (bar 3), and 2^nd^ devaluation test after second FI sessions/devalued condition (bar 4), respectively. Y-axis depicts normalized response rate which was obtained by dividing the numbers of lever press under non-devalued or devalued conditions with the response number on the previous day of the test session during the corresponding first 5 min period. The data of each individual were plotted with different colored dots. N = 10–12/each. Bar; MEAN ± S.D. * indicates significant differences between devalued vs. non-devalued conditions of the same group (*p* < 0.01), and † indicates a significant difference in devalued conditions between 1st vs. 2nd tests (*p* < 0.05).

Finally, when we examined whether there was any difference in the sensitivity to the outcome either before or after the second FI sessions in each group by conducting two-way ANOVAs for each group to compare between devalued and non-devalued conditions [*Test* (2: before and after the second FI sessions) × *Devaluation* (2: devalued vs. non-devalued)], we found some effects of *Devaluation*. For the FI + no cue and FI + non-contingent groups (n = 12/each), a significant main effect of *Devaluation* was revealed. (FI + no cue; *F*(1, 47) = 58.27, *p* < 0.01, partial **η**^2^ = 0.55, 95% CI = [0.35, 0.68], FI + non-contingent; *F*(1, 47) = 33.93, *p* < 0.01, partial **η**^2^ = 0.42, 95% CI = [0.20, 0.57]). Meanwhile, in the FI + contingent group (n = 10), a significant main effect of *Devaluation* was revealed (*F*(1, 39) = 20.23, *p* < 0.01, partial **η**^2^ = 0.34, 95% CI = [0.11, 0.52]). However, the interaction between *Test* and *Devaluation* was not significant (*F*(1, 39) = 4.63, *p* = 0.06). When we conducted a simple main effect analysis, we found that while the simple main effect of the *Devaluation* in the 1st test was significant (*F*(1, 18) = 22.25, *p* < 0.1, partial **η**^2^ = 0.55, 95% CI = [0.19, 0.72]), the simple main effect of the *Devaluation* in the 2nd test was not (*F*(1, 18) = 2.91, *p* = 0.11, partial **η**^2^ = 0.14, 95% CI = [0.0, 0.41]). In addition, the response rate in the devalued condition of the 2nd test was significantly higher than the that of the 1st test (*F*(1, 18) = 5.08, *p* = 0.03, partial **η**^2^ = 0.22, 95% CI = [0, 0.48]) (Fig. 4). Overall, we concluded that only the behavior of the FI + contingent cue group became habitual after the second FI sessions.

## Discussion

The present study showed consistent with our original assumption, cues provided during training under a FI 60-s schedule promoted habit expression in rats when they were contingent with the timing of reinforcement. As is well-known, attentional demand prevents both multitasking and automaticity^10^, which are characteristic daily hallmarks of habitual response^11,12^. Similarly, the significance of attention in goal-directed action has been implicated in habitual action^13,14^. However, to the best of our knowledge, few empirical reports, apart from a recent study by Thrailkill and colleagues^6^, have linked directly the significance of attentional burden with the transition from goal-directed action to the habitual response.

In the FI schedule, the subjects needed to estimate the precise elapsed time, such as 60 s, and attention resource allocation are required for this process^15,16^. It is widely accepted that many brain regions, including the striatum, are involved in this process^17^. In the case of striatal neurons, the neuronal activity gradually increases from a pressing start time to the next designated timing of the action of FI 60-s^18^. To date, there is no direct empirical attempt to prove the effectiveness of any distractor during the fixed timing under the FI schedule or a reversible and momentary disruption of incremental striatal activity during the same period. However, a pile of previous reports has demonstrated the difficulty of this process while performing a variety of dual-task paradigms^15,19–21^. Also, the longer the interval is, the wider the variance in subjective duration appears^22^. Thus, it is highly likely that the amount of sustained attention required for estimating an elapsed time is cognitively cost-consuming and critical for performing FI-like behavioral performance. In addition, the longer the intervals are, the more constant attentional allocation is demanded. Supporting this idea, many psychiatric disorders accompany attentional impairment and demonstrate the dysfunction of the estimation of elapsed time^23–25^. Moreover, the impaired performance of estimating interval timing in the animals with septal lesions can be rescued using external cues signaling the end of the required delay in differential reinforcement of low response rates schedules^26,27^. This implies that these cues might help to decrease the attentional load for this estimation, as demonstrated in this study.

As mentioned in the Introduction, it appears that the findings by Thrailkill and colleagues contradict those by DeRusso and colleagues in terms of uncertainty^5,6^. However, given highlighting an attention-based framework, the conflict between these two studies could be interpreted differentially. In contrast to the FI schedule, it is reasonable to consider that the RI schedule requires less sustained attention. For example, as demonstrated by DeRusso and colleagues^5^ as well as the data shown in Fig. 1, the subjects trained under FI 60-s withhold useless lever pressing for some time after the fixed timing to press the lever, but as the next reward timing approaches, they rush to press the lever. Thus, their performance of lever presses depicts a sigmoid- or scallop-like pattern. These data imply that their attention was maintained throughout the intervals to monitor the interval and press the lever on time. Conversely, in the RI schedule, the pattern of lever pressing becomes almost even^5^. A good example of an RI schedule-like situation in real life is a shooting-style video game in which targets appear unpredictably. Under such a scenario, a subject uses one hand to manipulate a joystick to change his or her location to target or hide in a goal-directed manner, which requires intensive and constant attention. Meanwhile, the other hand presses the shooting button in an almost random and automatic manner while paying little attention to whether their target is in sight or their target is hit. This strategy is more reliable for achieving high scores than paying constant attention to both shooting (action) and hit (outcome) contingencies, and it permits highly attention-demanding goal-directed actions concomitantly. Even when an instructive auditory cue is present in addition to the RI schedule, the subject has to momentarily pay attention to it, as observed in a typical stimulus-induced response. If the targets appear in the FI schedule, this game would be far more difficult to play.

Considering this attention-based advantage of the RI schedule, we aimed to minimize the essential attentional load for estimating a fixed elapsed time in the FI schedule, which is resistant to habit formation. In short, although the RI schedule, which is more habit-prone, is accompanied by a high degree of uncertainty, it demands less sustained attention. Contrarily, the FI schedule is linked to lower uncertainty. Thrailkill and colleagues claimed that when the reinforcer is predictable and the subject does not need to pay attention to the stimulus and action as proposed by the Pearce-Hall model^28^, then a habit will be developed^6^. Thus, this attentional demand depends on the computation of reward probability. Meanwhile, in the present study, we highlighted the significance of computation-independent attention which is necessary to be sustained for estimating a designated elapsed time in habit formation. As abovementioned, the FI schedule requires more sustained attention. In this FI 60-s schedule study, we provided instructive cues to animals to reduce their sustaining attentional load while leaving the uncertainty (or predictability) untouched, and this manipulation resulted in habit development. Thus, in addition to the uncertainty/predictability issue, we speculate that relief from sustained attention for estimating an elapsed time is also critical for promoting the goal-directed-to-habit transition. Consistent with this model, goal-directed action control is impaired in an animal model of attention-deficit/hyperactivity disorder that cannot sustain attention and in which habitual response predominates^29^ (also see ref. ^30^).

However, there is an alternative interpretation of our findings. Because devaluation effects are weakened when the context is extinguished before the devaluation phase^31,32^, it is also possible that the tone presented in the contingent group provided the animals with an opportunity to learn that the absence of the cue signals no reinforcement. In this scenario, it might be that the phasic tone, which is a better predictor of the reinforcement than the no-tone period, would modulate animals’ motivation. Unfortunately, we performed outcome devaluation tests in this study without presenting the auditory cues that were used during the training sessions; thus, we could not address the magnitude of the impact of such a process on the results.

The second limitation of this study is that we did not perform a contrasting experiment of the RI schedule in parallel for comparison. Because the findings illustrated that reward-contingent auditory cues required only 2 weeks for developing a habit, which was much faster than the 4 weeks required for the regular RI schedule in our previous study^7^, we speculate that the brief presentation of a stimulus contingent with the reward availability, such as light or sound, but not with lever pressing, promotes much faster habit expression than that observed under a typical RI schedule. Meanwhile, the cue contingent on lever pressing might delay the habit promotion, because it adds attentional demand, at least for a while.

Finally, the use of a single interval parameter limits the extent of our conclusions. As recently indicated by Dickinson and Perez, the action-outcome rate correlation in the framework of FI schedule-induced scallop-like pattern should be an important determinant of goal-directed control^33^. If the interval between rewards is shorter, this scallop-pattern-based rate correlation could not be recognized by animals, which may prevent goal-directed action. Additional studies are necessary to clarify these issues in the future.

In conclusion, our study showed that minimizing the temporal attentional load during operant learning under an FI schedule promoted the transition from goal-directed to habitual action. This result illustrates the significance of attentional processes in the balance of dual decision-making systems, goal-directed and habitual.

## Supplemenatry information

Supplemenatry information.
