# Supplementary material for: Sustaining temporal attention prevents habit expression during operant learning in rats
Source: Sci Rep. 2020 Jun 25;10:10303. doi: 10.1038/s41598-020-67304-y (PMC7316773; doi:10.1038/s41598-020-67304-y)

Title: Sustaining temporal attention prevents habit expression during operant learning in rats.

Author: Ziqiao Lin[1,2], Hiromi Nishikawa[1,2], Yoshio Iguchi[1,2,4], Akira Iwanami[5], Mitsuru Kikuchi[2,3], Shigenobu Toda[2,5,6]

1. Contributed equally to this study
2. Department of Psychiatry and Behavioral Science, Kanazawa University School of Medicine
3. Research Center for Child Mental Development, Kanazawa University
4. Department of Molecular Genetics, Institute of Biomedical Sciences, Fukushima Medical University
5. Department of Psychiatry, Showa University School of Medicine
6. Corresponding author

Supplemental information

Supp Fig. 1

The median of the timing of lever pressing increased gradually from FI30 to FI45 and to FI60 (one-way ANOVAs (*session timepoint* (FI30, FI45, FI60-Day1, FI60-Day7, FI60-Day14); $F(4, 175) = 134.8$, $p < .01$, partial $\eta^2 = 0.75$, 95% CI[0.69, 0.79] ; Supp Fig. 1A). The proportion of lever press at 25–30 s in total, that is the marker of the achievement of FI30, was decreased at the same time (one-way ANOVAs (*session timepoint* (FI30, FI45, FI60-Day1, FI60-Day7, FI60-Day14); $F(4, 175) = 111.3$, $p < .01$, partial $\eta^2 = 0.55$, 95% CI[0.45, 0.61] ;Supp Fig 1B). The results of Tukey's multiple comparisons tests showed that all comparisons were robustly significant ($p < .01$), apart from some comparisons of FI60 between Day1, Day 7, and Day14 (Supp Fig. 1A and B), indicating that the peak time of lever pressing changed from approximately 30 s to 45 s and 60 s as the designated interval increased.

Supp. Fig. 2

As the operant training under the FI60 schedule proceeded, the number of lever presses at a shorter interval (25–30 s) reduced, whereas the number of lever presses at longer intervals (40–45 s and 55–60 s) increased. Specifically, the interval between two consecutive lever presses lengthened [two-way ANOVA (*lever press timing* (3: 25-30 s, 40-45 s, 55-60 s) x *day* (3: FI60-Day1, 7, 14); *interaction*, $F(4, 315) = 65{,}674$, $p < .01$, partial $\eta^2 = 1.00$, 95% CI[1.00, 1.00]; *Day*, $F(2, 315) = 79{,}704$, $p < .01$, partial $\eta^2 = 1.00$, 95% CI[1.00, 1.00] ; *lever press timing*, $F(2, 315) = 1{,}621{,}624$, $p < .01$, partial $\eta^2 = 1.00$, 95% CI[1.00, 1.00]]. The results of Tukey's multiple comparisons tests showed that all comparisons were robustly significant ($p < .01$), apart from the comparison of a 40–45 s interval between Day1 and Day 7.

Supp. Fig. 3

One-way ANOVAs (*days* (Day 1, 4, 7, 10, 14)) revealed any significant alteration neither in the number of lever presses ($F(4, 175) = 1.90$, $p = .11$, partial $\eta^2 = 0.04$, 95% CI[0, 0.09] (panel A), in the number of pellets obtained, $F(4, 175) = 1.54$, $p = .19$, partial $\eta^2 = 0.03$, 95% CI[0, 0.08] (panel B), nor in the ratio of lever presses to pellets, $F(4, 175) = 1.33$, $p = .26$, partial $\eta^2 = 0.03$, 95% CI[0, 0.07] (panel C).

Supp. Fig. 4

Two-way ANOVAs (*group* (3: FI + no cue, FI + contingent cue, FI + non-contingent cue) x *day* (7: Day 15, 17, 19, 21, 23, 25, 28)) revealed that the number of lever presses (*interaction*, $F(12, 231) = 1.60$, $p = .09$, partial $\eta^2 = 0.08$, 95% CI[0, 0.10]; *day*, $F(6, 231) = 1.67$, $p = 0.13$, partial $\eta^2 = 0.04$, 95% CI[0, 0.08]; *group*, $F(2, 231) = 1.50$, $p = .23$, partial $\eta^2 = 0.01$, 95% CI[0, 0.05]), the number of pellets obtained (*interaction*, $F(12, 231) = 1.68$, $p = .07$, partial $\eta^2 = 0.08$, 95% CI[0, 0.11]; *day*, $F(6, 231) = 2.34$, $p = .03$, partial $\eta^2 = 0.06$, 95% CI[0, 0.10]; *group*, $F(2, 231) = 1.52$, $p = .22$, partial $\eta^2 = 0.01$, 95% CI[0, 0.05]), as well as the ratio of lever presses to pellets (*interaction,* $F(12, 231) = 1.72$, $p = .08$, partial $\eta^2 = 0.01$, 95% CI[0, 0.11]; *day*, $F(6, 231) = 1.21$, $p = .30$, partial $\eta^2 = 0.03$, 95% CI[0, 0.06] ; *group*, $F(2, 231) = 1.83$, $p = .16$, partial $\eta^2 = 0.02$, 95% CI[0, 0.06]), did not reach significance.

Supplemental figure legends

Supp. Fig 1

Operant training toward FI60 schedule affects the timing of lever press over sessions. A. The median of the timing of lever press (Y-axis) was increased according to the development of FI schedules. B. The proportion of lever press at 25-30 s in total (Y-axis) was decreased according to the development of FI schedules. N = 36/each. Bars: mean ± S.D.. N = 36/each. * indicates significant differences vs. FI30 ($p < .01$), † indicates a significant difference vs. F45 ($p < .01$), ‡ indicates significant differences vs. FI60-Day1 ($p < .01$), and § indicates significant differences vs. FI60-Day7 ($p < .01$).

Supp Fig. 2

As the session under FI60 schedule went on, the timing of lever press gradually became closer to around 55-60 s. N = /each. Bars: mean ± S.D.. * indicates significant differences vs. 25-20s/Day1 ($p < .01$), † indicates a significant difference vs. 40-45s/Day1 ($p < .01$), ‡ indicates significant differences vs. 55-60s/Day1 ($p < .01$), § indicates significant differences vs. 25-20s/Day7 ($p < .01$), ¶ indicates a significant difference vs. 40-45s/Day7 ($p < .01$), and # indicates a significant difference vs. 55-60s /Day7 ($p < .01$).

Supp Fig 3.

Characterization of the consequences of 14-days operant learning with FI60 schedules (N=36). A. Total number of lever press. B. Total number of obtained pellet. C. The ratio of total number of lever press to the total number of obtained pellet. X axis represents day number of total FI sessions from the beginning. Bar: mean ± S.D..

Supp Fig 4.

Characterization of the consequences of 14-days operant learning with distinct FI60 schedules. A. Total number of lever press. B. Total number of the obtained pellet. C. The ratio of total number of lever press to the total number of the obtained pellet. N=12/each. X-axis represents the day number of total FI sessions from the beginning. Bar; mean ± S.D..

Supplement Fig 1

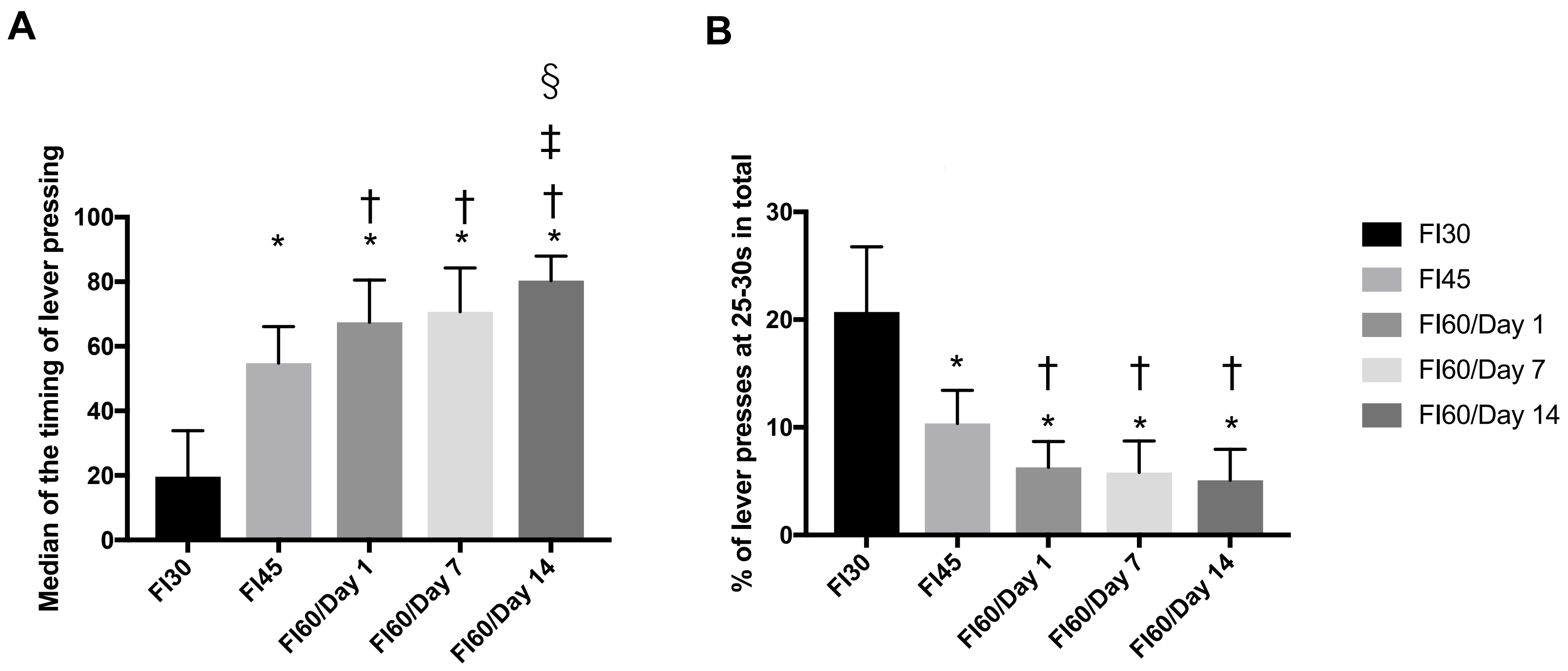

Supplement Fig 2

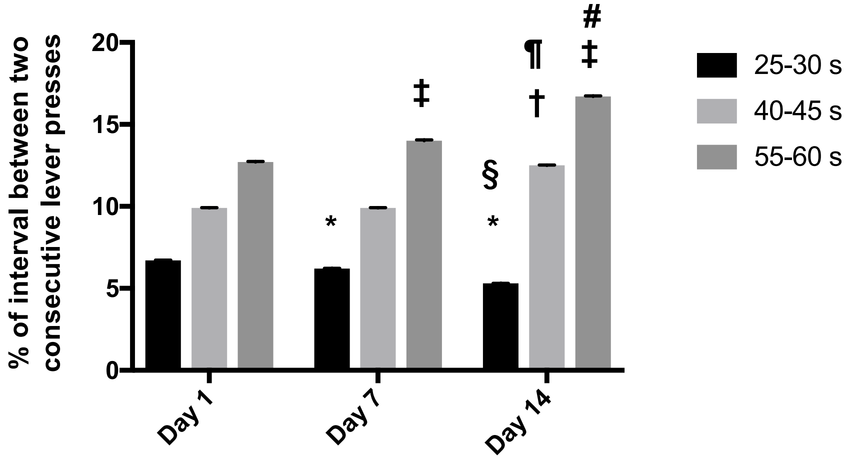

Supplement Figure 3

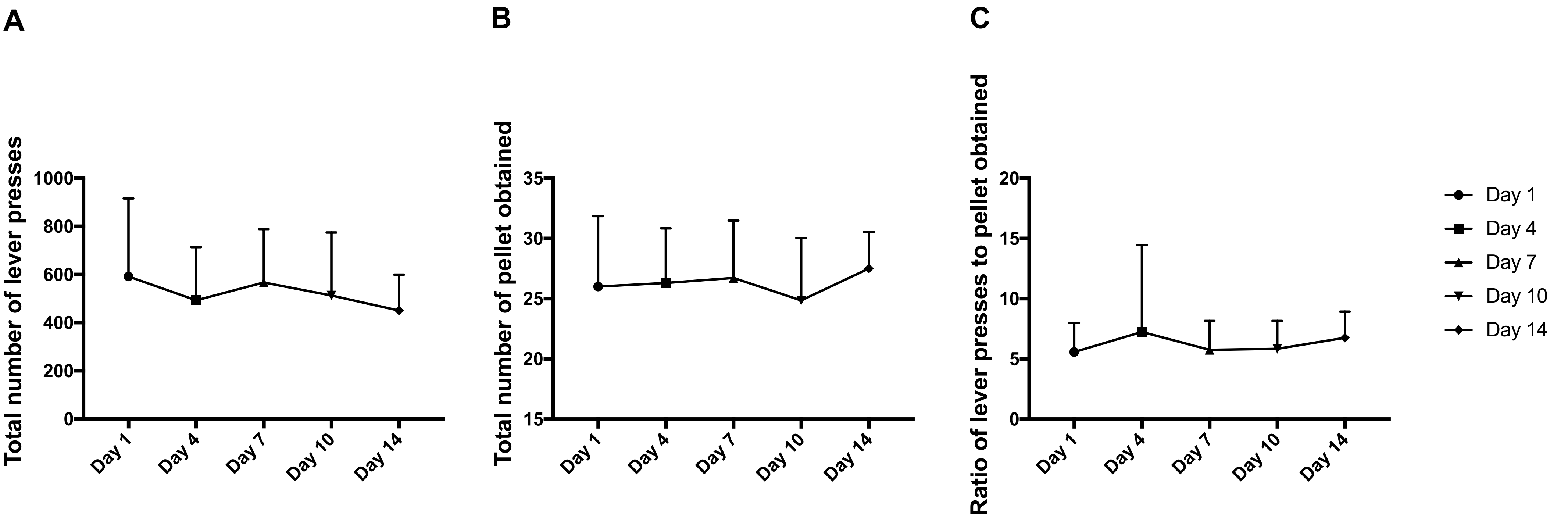

Supplement Figure 4

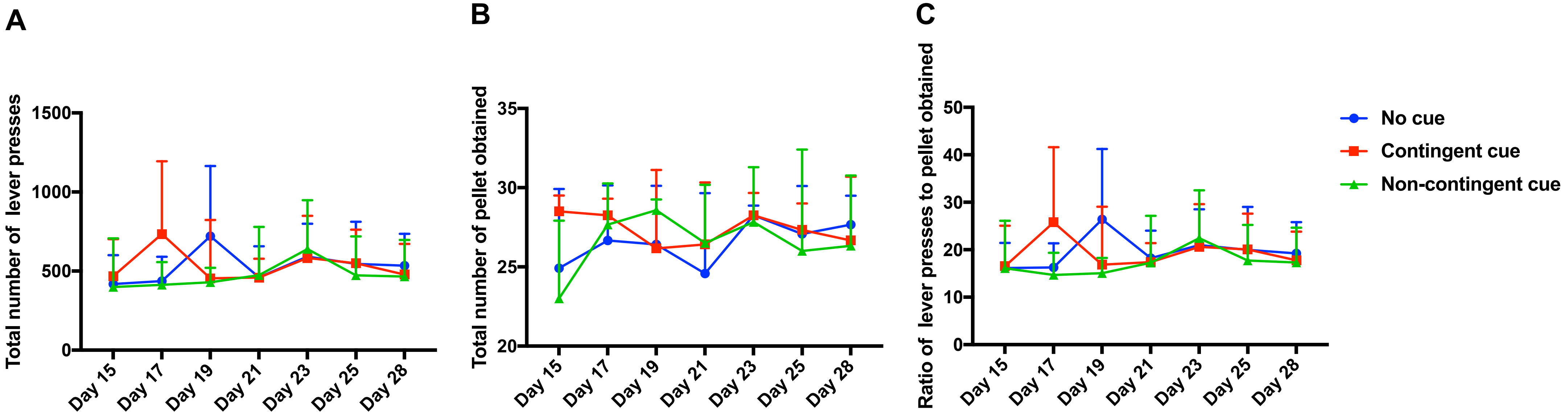

Supplement: Supplementary file 1 — Supplemenatry information. [file 41598_2020_67304_MOESM1_ESM.pdf]
